# Knowledge and utilization of contraceptive devices among unmarried undergraduate students of a tertiary institution in Kano State, Nigeria 2016

**DOI:** 10.11604/pamj.2017.26.103.11436

**Published:** 2017-02-28

**Authors:** Zainab Datti Ahmed, Ibrahim Baffa Sule, Mohammed Lukman Abolaji, Yahaya Mohammed, Patrick Nguku

**Affiliations:** 1 Aminu Kano Teaching Hospital, Kano, Nigeria; 2 Bayero University Kano, Kano, Nigeria; 3 Nigeria Field Epidemiology and Laboratory Training Program, Nigeria; 4 Usman Danfodiyo University Sokoto, Sokoto, Nigeria

**Keywords:** Determinants, contraceptives use, unmarried, undergraduates, Bayero University Kano

## Abstract

**Introduction:**

Students in the universities mostly live independently from their parents or guardians, some of them for the first time. This gives them freedom and opportunity for high risk behavior such as unplanned and unprotected sex. The results of such sexual experimentation may include unplanned and or unwanted pregnancies that may lead to unsafe abortions and sexually transmitted infections (STIs) including HIV/AIDS. Contraception has the potential to prevent unwanted pregnancies, abortion, and STIs. This study aimed at assessing the general knowledge on contraceptives, sexual practices, and level of utilization of contraceptives devices among unmarried students of the Bayero University Kano.

**Methods:**

We did an institutional based cross-sectional descriptive study. We administered a pretested, self-administered, structured questionnaire to randomly selected unmarried undergraduate students of the institution. We analyzed data using Microsoft Excel 2016 and Epi-info7.

**Results:**

A total of 300 students were interviewed. The median age for respondents was 23 years with an age range of 16–25 years. Male respondents made up 61.3% (184) while the females made up the remaining 38.7% (116). Also, 158(47.33%) of respondents lived outside the school campus, while 158(52.67%) lived in the school hostels. Knowledge on contraception was 87.7% among respondents with internet (91%) and media (89.3%) as the commonest sources of knowledge. Proportion of sexually active students was 10.67%, while prevalence of contraceptive utilization among sexually active students was 15.63%. About 8(25%) had their sexual debut at < 16years of age, 22(68.75%) at ages between 16-20years, and 4(12.5%) above 20years of age. All sexually active respondents practice vaginal sex. Most sexual debuts were planned (44.75 %) and with friends (86.4%), and they occurred between the ages of 16-20years age group in 70.3% of respondents.

**Conclusion:**

Even though knowledge on contraceptive used was high among the respondents, utilization of contraceptives among sexually active students was low, thus creating a window for possible unintended and unwanted pregnancies among these group of students.

## Introduction

Globally, youths are more sexually active than any subgroup of the population [1]. The proportion of sexually active adolescents has been on the increase worldwide including Nigeria [1–3]. This is exposing large numbers of youths to the risk of unwanted pregnancies and sexually transmitted diseases (STDs) including HIV/AIDS. The bio-social gap between the early onset of puberty and the increasing age of marriage has widened in most African countries [4]. This widens the window for pre-marital sexual activity that further exposes them to the risk of unwanted pregnancies, unsafe abortions, and sexually-transmitted infections (STIs) [1, 4]. Tertiary education gives students opportunity for greater independence from home, with new friendships and romantic or sexual relationships [5]. These new opportunities and freedom come with high risk behaviors such as unplanned and unprotected sex sometimes with multiple sexual partners [3–5]. These new behaviors are often hinged on curiosity, peer pressure and sexual maturation. The result of such sexual experimentation includes unplanned and or unwanted pregnancies that leads to abortions, (mostly unsafe), and sexually transmitted infections including HIV/AIDS [6, 7]. The level of prostitution and all forms of promiscuous behavior associated with students of tertiary institutions and other anti-social sexual behaviors, coupled with the widespread of sexually transmitted infections and teenage pregnancy amongst undergraduates is of great concern to the society [8]. The absence of contraceptive services in tertiary institutions has further led to increased rates of unwanted pregnancy, unsafe abortions and STIs among undergraduates of these institutions [8, 9]. Contraception is defined as deliberate prevention of conception or impregnations [10, 11]. Numerous methods of contraception are available hence classification varies [12]. Abstinence provides 100 percent protection from HIV, STIs, and pregnancy. For some, this means avoiding vaginal, anal, and oral-genital intercourse altogether. Other types of contraception include; Barrier methods (condoms), hormonal methods (Pills), Implants, Intra-Uterine Contraceptive Devices (IUCDs) and Emergency contraception [13]. This study aimed at assessing the general knowledge on and level of utilization of contraceptives, and determine factors that affect the use of contraceptive devices among unmarried students of the Bayero University Kano. And, to also highlight some of the attributes and practices of the students with regards sexual behaviors.

## Methods

**Study settings:** Bayero University Kano (BUK) was established in October 1960. It is located in Kano city, Nigeria. The University has two campuses (old and new campuses) and an affiliated teaching hospital. Bayero University Kano has a total of about 24,033 undergraduate students, 16,405 males and 7,628 females (University registrar’s records). The BUK student’s composition represents all states in Nigeria and the two major religions [14].

**Study population:** This study was conducted among both male and female unmarried (never married before, divorcees and widowers) undergraduate students of Bayero University Kano, who have spent at least one complete session.

**Study design:** We did a Cross-sectional descriptive study. Sampling a total of 300 undergraduate students.

**Inclusion criteria**: Student must have stayed one complete session in the school; student must be officially registered in the school; student must be between the ages of 16 and 25years.

**Exclusion criteria**: Married students; medical students in the clinical section.

**Sample size determination:** The minimum sample size was obtained using the formula for cross-sectional study designs.

N = Z^2^pq d^2^

Where: N= Minimum sample size; Z= the standard normal deviation corresponding to 95% levels of significance (1.96); P= prevalence of sexually active unmarried undergraduate students (77.6% or 0.776) [3].

q = 1-p = 0.224

d = degree of precision = 0.05

N = (1.96) ^2^ x 0.776 x 0.224 = 267 (0.05)^2^

We added 10% of this value to cover for attrition and non-responses, giving a value of 294. We then rounded it up to 300 students as the minimum sample size.

**Sampling technique:** A multistage sampling technique was done. Eight (8) faculties were randomly selected from the 15 faculties of the university. In the second stage, one department was also randomly selected from each of the eight selected faculties using simple random sampling. In the third stage, a sample of students proportionate to their number in each level was systematically selected from the sampled departments until sample size was attained. Anonymous self-completed questionnaires were administered to those who provide informed consent. The survey was conducted during a regular semester session.

**Study instrument:** A pretested self-administered questionnaires were administered to each participating student to obtain relevant information.

**Data analysis:** We collected and analyzed data using Microsoft Excel 2016 and Epi Info7.2. Absolute numbers and simple percentages were used to describe categorical variables. Quantitative variables were described using measuring of central tendency (mean, median and standard deviation) and proportions.

**Ethical issues:** Ethical clearance was obtained from Kano State Ministry of Health ethical committee. Privacy and confidentiality were maintained both during and after the administration of the questionnaire by ensuring that the filled questionnaires are dropped into a box containing other questionnaires.

## Results

Of the 320 questionnaires that were distributed, 306(95.6%) were returned as filled questionnaires. Six (1.9%) of these were wrongly filled. Thus, a total of 300(93.8%) eligibly filled questionnaires were retrieved. The median age for respondents was 23years with an age range of 16 – 25years. The average age of respondents was 22years (SD±4years). Majority of respondents 246(82%) were within the 21-25years age group, while the rest 54(18%) were in the 16-20years age group. Male respondents made up 61.3%(184) while the females made up the remaining 38.7%(116) of respondents. Islam was the main religion of respondents with 229(76.3%), followed by Christianity 68(22.7%) and others 3(1%). Most of the respondents were of the Hausa tribe with 165(55%), followed by Yoruba 78(26%), Igbo 38(12.67%) and others 19(6.33%). Among responding students, 158(47.33%) lived outside the school campus, slightly lower than those living within the Hostels 158(52.67%) (Table 1). Of the 300 respondents, 32(10.67%) have had sexual intercourse prior to the study, and of the 32 sexually active respondents, 8(25%) had their first intercourse at < 16years of age, while 22(68.75%) had their first intercourse at ages between 16-20years, and 4(12.5%) above 20years of age. All sexually active respondents practiced vaginal sex. Only 5(15.63%) of the sexually active respondents regularly used any form of contraceptives, while 8(25%) occasionally used contraceptives. Majority 19(59.37%) of the sexually active respondents had never used any form of contraceptives. Most (43.75%) intercourse among sexually active respondents were planned, while curiosity and pleasing of partners equally accounted for the rest (Table 2). Majority of respondents 263(87.67%) defined contraceptive use as prevention of pregnancy, while 28(9.33%) defined it as termination of pregnancy and 4(1.33%) as improving sexual capacity. When respondents were asked on types of contraceptive devices they knew, 294(98%) named condoms, 282(98%) pills, 247(82.33%) injectable, 147(49%) implants, 181(60.33%) IUCDs, and 250(83.33%) abstinence. Only 2(0.67%) had no knowledge of any type of contraceptives. Media, internet, and peers were equally the sources of knowledge on contraceptives with 93(31%) each. Parents (3%) and Health workers (4%) were the other sources of knowledge on contraceptives (Table 3).

**Table 1:** Socio-demographic characteristics of responding undergraduate students of Bayero University Kano, Nigeria 2016

Age Group	Frequency (n=300)	Percentage
16 - 20	54	18.00
21 - 25	246	82.00
**Sex**		
Male	184	61.30
Female	116	38.70
**Religion**		
Islam	229	76.30
Christianity	68	22.70
Others	3	1.00
**Ethnicity**		
Hausa	165	55.00
Yoruba	78	26.00
Igbo	38	12.67
Others	19	6.33
**Place of Resident**		
Hostel	158	52.67
Off Campus	142	47.33

**Table 2:** Sexual characteristics and use of contraceptive devices among undergraduate students of Bayero University Kano, Nigeria 2016

Sexual activity	Frequency (n=300)	Percentage
Has had intercourse	32	10.67
Never had intercourse	216	72.00
Refused to say	52	17.33
**Age at first intercourse**	**Frequency (n=32)**	
<16	8	25.00
16 - 20	22	68.75
21 - 25	4	12.50
**Type of intercourse**		
Vaginal sex	32	100.00
Anal sex	0	0.00
Both	0	0.00
**Use of Contraceptives**		
Regular user	5	15.63
Occasional user	8	25.00
Never used	19	59.37
**Reason for intercourse**		
Planned	14	43.75
Curiosity	9	28.13
To please partner	9	28.12

**Table 3:** Knowledge of contraceptives among undergraduate students of Bayero University Kano, Nigeria 2016

Knowledge	Frequency (n=300)	Percentage
**Definition of contraceptive**		
Prevention of pregnancy	263	87.67
Termination of pregnancy	28	9.33
Improving sexual capacity	4	1.33
Don’t know	5	1.67
**Types of contraceptives known**		
Condoms	294	98.00
Pills	282	94.00
Injectable	247	82.33
Implants	147	49.00
IUCDs	181	60.33
Abstinence	250	83.33
Don’t know	2	0.67
**Source of contraceptive Knowledge**		
Media	93	31.00
Internet	93	31.00
Peers	93	31.00
Parents	9	3.00
Health worker	12	4.00

## Discussion

Our study found the knowledge on contraceptive methods to be high (87.67%) among undergraduate students of Bayero University Kano. This finding is consistent with similar study done in Enugu State, Southeastern Nigeria in 2005, which put awareness of contraception among undergraduate students at 95% [15]. But, it contradicts the result of a similar study done in a tertiary institution of Osun State, Southwestern Nigeria, which demonstrated knowledge on contraceptive use to be 19.7% among undergraduate students of the institution [7]. Knowledge and awareness of contraceptives among the students was not related to their sexual practices. This high level of awareness can be attributed to increase use of social network/media, which is one of the main source of knowledge as opined by this study, and is consistent with other similar studies which identified the internet and social media as the highest sources of health-related information to undergraduate students [5]. Among the contraceptive methods, knowledge on male condoms was the highest among the students followed by oral contraceptive pills and the injectable. These findings were consistent with the findings of similar studies conducted in 2003 and 2009 [3, 5]. Despite the high knowledge on contraceptive methods among the students, the utilization of contraceptives among sexually active students was very low. We found the proportion of sexually active students to be low as compared to similar studies done in 2011 at a high institution in southwestern, Nigeria [1]. But, this finding is inconsistent with what was obtained in a similar study done at another institution of Southwestern Nigeria which found the proportion of sexually active undergraduate to be 63% [16]. The level of regular utilization of contraceptives among sexually active undergraduates found in this study was low (15.63%) when compared to similar studies conducted in 2009 and 2014 which put the percentage utilization of contraceptives among sexually active undergraduates at 25.4% and 32.6% respectively [3, 7]. The low utilization of contraceptives found in this study could be attributed to cultural and religious factors which may have affected the students responds to the questions. Our study also showed sexual debut to be more in the age range of 21-25 years which appears to be more delayed compared to the 11-17 years and 15-19 years found in similar studies conducted in southwestern part of Nigeria [1]. This may be due to the average age of admission at Bayero University, which is around 20years. Notwithstanding, most sexual debut were planned and fewer are done to please the other partner.

## Conclusion

Despite a high level of knowledge and awareness on contraceptive us among undergraduate students, and low proportion of sexually active students, the level of utilization of contraceptives among the students was low. Looking at the wider picture, this is a serious cause for concern, and will require concerted efforts by the society, the university authorities and parents of the students in order to tackle it. **Recommendations:** The university authority should provide adolescent health clinics which will provide students with counseling on sex related matters and also provide easily accessible contraceptive services where the need arises. Enlightenment of students on the dangers and consequences of unprotected sex. The commonest source of information for the students, which is the social media, should be exploited and utilized in disseminating this information. However, total abstinence should be encouraged among all students. **Limitations to the study**: Only students who have completed a session were selected for the study, hence this has excluded a substantial number of eligible undergraduates in their first year of study. The generalization of the research in those in the first year. Self-administered questionnaires are subject to information bias, and since the study touches on sensitive issues, the possibility of concealment by the students cannot be excluded.

### What is known about this topic

- Majority of university undergraduates experience wider independence for the first time while on campus;
- Most high risk sexual behaviors were initiated while in the university.

### What this study adds

- The level of awareness and practice of risky sexual habits of undergraduate in a Northwestern tertiary institution of Nigeria;
- Utilization of contraceptives among these students.
